# Conversion of Food Waste into 2,3-Butanediol via Thermophilic Fermentation: Effects of Carbohydrate Content and Nutrient Supplementation

**DOI:** 10.3390/foods11020169

**Published:** 2022-01-10

**Authors:** Dajun Yu, Joshua O’Hair, Nicholas Poe, Qing Jin, Sophia Pinton, Yanhong He, Haibo Huang

**Affiliations:** 1 Department of Food Science and Technology, Virginia Polytechnic Institute and State University, 1230 Washington St. SW, Blacksburg, VA 24061, USA; dajunyu@vt.edu (D.Y.); johair@tnstate.edu (J.O.); pnick1@vt.edu (N.P.); jin622@vt.edu (Q.J.); spinton@ucdavis.edu (S.P.); yanhong6@vt.edu (Y.H.); 2 Department of Biological Sciences, Tennessee State University, 3500 John A Merritt Blvd, Nashville, TN 37209, USA

**Keywords:** food waste, 2,3-butanediol, carbohydrate, nutrient, *Bacillus licheniformis*, thermophilic fermentation

## Abstract

Fermentation of food waste into 2,3-butanediol (2,3-BDO), a high-value chemical, is environmentally sustainable and an inexpensive method to recycle waste. Compared to traditional mesophilic fermentation, thermophilic fermentation can inhibit the growth of contaminant bacteria, thereby improving the success of food waste fermentation. However, the effects of sugar and nutrient concentrations in thermophilic food waste fermentations are currently unclear. Here, we investigated the effects of sugar and nutrients (yeast extract (YE) and peptone) concentrations on 2,3-BDO production from fermenting glucose and food waste media using the newly isolated thermophilic *Bacillus licheniformis* YNP5-TSU. When glucose media was used, fermentation was greatly affected by sugar and nutrient concentrations: excessive glucose (>70 g/L) slowed down the fermentation and low nutrients (2 g/L YE and 1 g/L peptone) caused fermentation failure. However, when food waste media were used with low nutrient addition, the bacteria consumed all 57.8 g/L sugars within 24 h and produced 24.2 g/L 2,3-BDO, equivalent to a fermentation yield of 0.42 g/g. An increase in initial sugar content (72.9 g/L) led to a higher 2,3-BDO titer of 36.7 g/L with a nearly theoretical yield of 0.47 g/g. These findings may provide fundamental knowledge for designing cost-effective food waste fermentation to produce 2,3-BDO.

## 1. Introduction

Food waste and loss pose a severe threat to the sustainability of our food system. Globally, an average person discards 65 kg of food each year, totaling 1.3 billion kg of food lost annually [1,2]. In the United States, approximately 40% of the food supply becomes waste, and most of it ends up in landfill, resulting in huge economic loss and significant emission of greenhouse gases [3]. Developing novel ways to convert food waste to value-added products is a promising approach to reduce global food waste and promote environmental sustainability. Food waste has been considered a good substrate for various renewable chemicals such as ethanol, butanol, and lactic acid [4,5,6,7,8], as it usually contains a high amount of carbohydrates (e.g., sugar, starch). Meanwhile, food waste also contains substantial amounts of proteins, vitamins, and minerals [9], which support the growth and metabolism of microorganisms in fermentation processes.

2,3-butanediol (2,3-BDO) is a promising commodity chemical with a wide range of applications [10,11]. Being odorless, colorless, and transparent at ambient temperature, 2,3-BDO can be used to make antifreeze agents, synthetic rubber, perfumes, printing inks, plasticizers, explosives, and pharmaceutical carriers [12,13,14,15]. 2,3-BDO can be synthesized in a variety of ways. Currently, chemical synthesis followed by purification is the most common way to produce 2,3-BDO, but it is a costly and complex process with multiple phases [15,16]. Additionally, with the depletion of non-renewable petroleum resources, efforts have been made to investigate the microbial synthesis of 2,3-BDO [17]. Many mesophilic strains have been reported to function well in the synthesis of 2,3-BDO via sugar fermentation. The gram-negative leading producers include *Klebsiella pneumoniae*, *Klebsiella oxytoca*, *Enterobacter aerogenes*, *Enterobacter cloace*, and *Serratia marcescens* [18]. The gram-positive leading hosts include *Bacillus subtilis*, *Bacillus licheniformis*, *Bacillus amyloliquefaciens* and *Paenibacillus polymyxa*. Using food waste for mesophilic fermentation at 30 to 40 °C creates concerns for a wide range of contamination and requires strict sterilization of fermentation media. Unfortunately, steam sterilization, the most common method, is energy intensive [19,20]. Furthermore, the harsh sterile environment may result in significant nutrient loss and the production of toxic compounds, thus jeopardizing the fermentation processes [21]. To solve this situation, much work has been conducted to isolate thermophilic strains that can thrive at temperatures ranging from 50 to 60 °C, where mesophilic microbes cannot reproduce. As a result, it can reduce the risk of cross-contamination during fermentation and potentially allow non-sterile fermentation [22,23]. Xiao et al. [21] used a thermophilic bacteria, *Geobacillus* sp. XT15 for the production of acetoin and 2,3-BDO, with an optimum growth temperature between 45 and 55 °C. Wang et al. [24] reported that *B. licheniformis* BL5 and BL8 showed substantial 2,3-BDO yield and productivity (0.73 mol/mol for BL8 in xylose and 0.9 mol/mol for BL5 and BL8 in glucose) under the temperature range of 50 to 55 °C. However, in these studies, relatively pure sugars (e.g., glucose and mixed sugars from lignocellulose hydrolysis) were used as feedstock for thermophilic fermentations. Their fermentation performance using food waste, which is much more complex, has yet to be studied.

Recently, we have isolated a new thermophilic *B. licheniformis* strain, YNP5-TSU, from the hot springs in Yellowstone National Park (lat—44.7803, long—110.6981) (#YELL-2015-SCI-6074) [20]. Compared with other reported thermophilic strains, this strain can also carry out fermentation at alkaliphilic conditions at or below an initial pH of 9.0. Since most of the contaminating bacteria that occur in fermentation are acidophilic (e.g., *Lactobacillus* spp., *Acetobacter* spp.) [25,26], the unique combination of thermophilic and alkaliphilic properties of *B. licheniformis* YNP5-TSU allows this strain to out-compete most contaminating microorganisms without the use of sterilization [1]. This strain showed great performance in utilizing various sugar sources to produce 2,3-BDO with a high yield of 0.46 g/g at the optimal temperature from 50 to 55 °C [14]. However, in our previous study [14], a high amount of nitrogen nutrients (5 g/L peptone and 10 g/L yeast extract) were added to the glucose fermentation substrate to secure the growth and metabolism of *B. licheniformis* YNP5-TSU. The high amount of the costly nutrients increases the fermentation cost to produce 2,3-BDO and potentially inhibits its future commercialization at an industrial scale. Thus, it is important to investigate if the fermentation can be conducted at low-nutrient levels when pure glucose and food waste feedstocks are used as fermentation substrates. Moreover, high-solid fermentation is always desired because it can potentially increase the end-product concentration in the fermentation broth and may reduce the capital and operating costs of fermentation and product separation [4]. Conversely, previous researchers have indicated that a high initial sugar concentration can inhibit the 2,3-BDO fermentation due to the high osmotic pressure on microorganisms [27,28]. Therefore, the objective of this study is to investigate the effects of nutrients and sugar concentration on *B. licheniformis* YNP5-TSU fermentation using glucose- and food waste-based media, respectively. The findings of this study will assist in better understanding the thermophilic and alkaliphilic fermentation to produce 2,3-BDO and will help design a more economically friendly fermentation process using food waste.

## 2. Materials and Methods

### 2.1. Enzymes and Reagents

The enzymes used in this study are α-amylase (Liquozyme SC) and glucoamylase (Spirizyme Fuel), kindly provided by the Novozymes (Franklinton, NC, USA). The α-amylase and glucoamylase have a declared activity of 120 KNU-S/g, 750 AUG/g, and a density of 1.25 and 1.15 g/mL, respectively. The yeast extract used in this study is from Bacto-Dickinson & Co. (Sparks, MD, USA) and glucose standards were purchased from Sigma-Aldrich (St. Louis, MO, USA). Other reagents and chemicals were obtained from Fisher Scientific (Fair Lawn, NJ, USA).

### 2.2. Food Waste Collection, Pretreatment, and Hydrolysis

Food waste used in this study was collected from the campus food preparation center at Virginia Tech (Blacksburg, VA, USA). Its main components are bakery waste and some vegetables and fruits. After being homogenized by a blender, the food waste was stored under −20 °C until use. The chemical composition of the food waste was analyzed in our previous study [7] and briefly, the food waste consisted of 23.9% of starch, 7.8% of protein, 7.7% of soluble sugars, 3.2% of fat, 2.8% of neutral detergent fiber, 1.4% of ash, and 47.5% of moisture based on the wet matter basis.

Hydrolysis of food waste was carried out using Liquozyme SC to break down starch to dextrins and saccharification (using Spirizyme Fuel) to convert dextrins into reducing sugars. The optimal condition for starch hydrolysis of food waste was carried out using 0.01% (*w*/*w*) Liquozyme SC and 0.06% (*w*/*w*) Spirizyme Fuel, identified by the study of Jin et al. (2020) [7]. Starch hydrolysis, in detail, began by adding 703.9 g deionized water with 202.5 g food waste in a 2 L volumetric flask to achieve a solid loading of 13.3%. The pH of the food waste slurry was adjusted to 5.8 using 5 N sulfuric acid and the Liquozyme SC was added at a loading of 0.01% (*w*/*w*) based on the dry weight of the food waste. Afterward, the slurry was incubated for 2 h at 85 °C and 120 rpm in a shaking water bath. Upon completing liquefaction, the liquefied sample was cooled down to room temperature and the pH was adjusted to the optimal pH of Spirizyme Fuel at 4.2. Spirizyme Fuel was then added at a loading of 0.06% (*w*/*w*) of the dry weight of food waste and the mixture was incubated in the same water bath for 24 h at 65 °C with a shaking speed of 120 rpm. After saccharification, the slurry was centrifuged under 4 °C for 10 min at 16,639× *g*. The supernatant was filtered using Whatman #3 filter paper to further remove the suspended solid residues.

### 2.3. Initial Glucose Concentrations and Their Effects on Glucose Fermentation

*B. licheniformis* YNP5-TSU cultures (1 mL per vial) were stored in 20% glycerol under −80 °C and thawed to room temperature before using. A two-stage (P_1_ and P_2_) culturing method was used to prepare the *B. licheniformis* YNP5-TSU seed culture. To be detailed, the P_1_ growth media contained 60 g/L glucose, 10 g/L yeast extract, and 5 g/L peptone. The P_2_ growth media contained 40 g/L glucose, 10 g/L yeast extract, and 5 g/L peptone. Both P_1_ and P_2_ growth media were adjusted to pH 8.0 using 1N NaOH or 1N HCl solution. To inoculate, 1 mL of thawed inoculum was added to 100 mL of the P_1_ media and incubated in a shaking incubator (New Brunswick Scientific Inc., Edison, NJ, USA) at 50 °C for 18 h with a shaking speed of 150 rpm. After P_1_ inoculation, 20 mL of the culture was collected and transferred to the P_2_ media which was then incubated at 150 rpm at 50 °C until the OD_600_ (optical density) reached 1.0.

Fermentation media was prepared such that three glucose concentrations (40, 60, and 80 g/L) could be examined. Each was supplemented with 10 g/L yeast extract and 5 g/L peptone and adjusted to a pH of 8.0 using 1 N NaOH [14]. Then, 2.5 mL of the prepared P_2_ culture was inoculated into 47.5 mL of each glucose media (5% inoculation rate) in 150 mL baffled flasks. The fermentation was conducted in a shaking incubator at 50 °C at 150 rpm for 48 to 96 h depending on the different initial glucose concentrations. During fermentation, 1 mL samples were taken at 12 h intervals for the analysis of sugar and fermentation metabolites.

### 2.4. Yeast Extract and Peptone Concentrations and Their Effects on Glucose and Food Waste Fermentation

To investigate the effects of the supplemented nutrients on 2,3-BDO fermentation using glucose media and food waste hydrolysate media, fermentation was conducted at reduced nutrient levels. Both glucose media containing 60 g/L glucose and food waste hydrolysate media containing 60 g/L total sugars were prepared with gradually reduced yeast extract and peptone concentrations: (1) 10 g/L yeast extract and 5 g/L peptone, (2) 5 g/L yeast extract, and 2.5 g/L peptone, and (3) 2 g/L yeast extract and 1 g/L peptone. Glucose and food waste hydrolysate media were respectively inoculated with the prepared P_2_ culture at a 5% inoculation rate. The fermentation was operated at 50 °C with a shaking speed of 150 rpm. Unless there was a tendency of slow sugar consumption or no sugar being consumed, samples were obtained every 12 h until no sugars remained.

### 2.5. Initial Sugar Concentrations and Their Effects on Food Waste Hydrolysate Fermentation

Food waste hydrolysate was first diluted to total sugar concentrations of 40, 60, and 80 g/L, respectively. Then, 2 g/L of yeast extract and 1 g/L of peptone were added. Inoculation was similar to the protocol already mentioned and from this, the P_2_ culture was added to the food waste hydrolysate media at an inoculation rate of 5% and the fermentation was conducted in a shaking incubator at 50 °C at 150 rpm. Samples (1 mL) of the fermentation media were taken every 12 h until all sugars were consumed.

### 2.6. Fermentation Sample Analysis

All collected fermentation samples were centrifuged at 16,639× *g* (Model 5424, Eppendorf Inc., Hamburg, Germany) and the collected supernatant was filtered through a 0.20 µm syringe filter. Then, 200 µL of the supernatant was diluted 5 times with 0.005 M H_2_SO_4_. Glucose, fructose, and other fermentation metabolites including lactic acid, acetic acid, ethanol, and 2,3-BDO in the samples were quantified using high-performance liquid chromatography (HPLC) equipped with a refractive index detector (RID) (Agilent Technologies 1260, Santa Clara, CA, USA). Bio-Rad Aminex^®^ HPX-87H column (Bio-Rad Laboratories, Hercules, CA, USA) was used to separate different compounds using 0.005 M H_2_SO_4_ as the mobile phase with a flow rate of 0.6 mL/min and injection volume of 20 μL. The column temperature was set at 50 °C, and the total run time was 45 min per sample. The yield and fermentation rate of 2,3-BDO were calculated to evaluate the fermentation efficiency:

(1) $$2,3\text{-}\mathrm{BDO}\;\mathrm{yield}=\frac{Total\;2,3\text{-}BDO\;concentration\;in\;fermentation\;broth\;\left(g/L\right)}{Total\;sugar\;consumed\;in\;fermentation\;broth\;\left(g/L\right)}$$

(2) $$\mathrm{Glucose}\;\mathrm{consumption}\;\mathrm{rate}=\frac{Total\;sugar\;consumed\;in\;fermentation\;broth\;\left(g/L\right)}{Fermentation\;time\;\left(h\right)}$$

(3) $$2,3\text{-}\mathrm{BDO}\;\mathrm{productivity}=\;\frac{Total\;2,3\text{-}BDO\;concentration\;in\;fermentation\;broth\;\left(g/L\right)}{Fermentation\;time\;\left(h\right)}$$

### 2.7. Statistical Analysis

All fermentations were conducted in duplicate, and the results were presented as mean values (*n* = 2). One-way ANOVA analysis with Tukey’s Honestly Significant Difference (Tukey’s HSD) was conducted using SPSS (22.0.0.0, IBM Corporation, Armonk, NY, USA) to test if there are significant differences among different treatments. The *p* value was set as 0.05.

## 3. Results and Discussion

### 3.1. Fermentation on Glucose Media with Different Initial Glucose Concentrations

**Figure 1:**
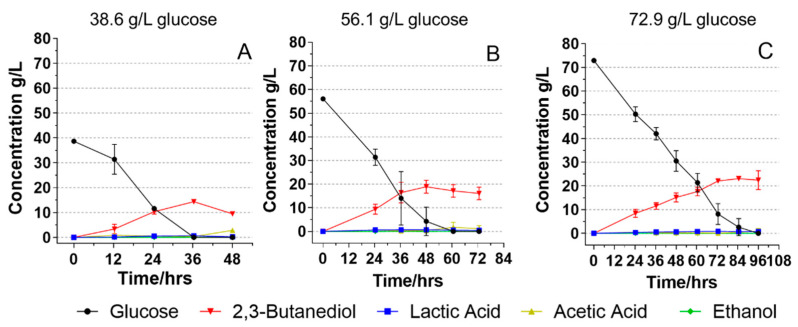
Fermentation of *B. licheniformis* YNP5-TSU using glucose fermentation media with three different initial glucose concentrations of 38.6 g/L (**A**), 56.1 g/L (**B**), and 72.9 g/L (**C**).

Due to the dilution effects of adding nutrient solutions and culture inoculum to the media, the actual initial glucose concentrations were 38.6, 56.1, and 72.9 g/L, respectively, as measured by HPLC. For the fermentation with an initial glucose concentration of 38.6 g/L (Figure 1A), it took 36 h for *B. licheniformis* YNP5-TSU to consume all glucose with the highest 2,3-BDO titer of 14.3 g/L and the corresponding 2,3-BDO yield of 0.37 g/g. However, after 36 h when all glucose was consumed, the YNP5-TSU started to consume the produced 2,3-BDO and produced 2.79 g/L acetic acid at 48 h. The conversion of 2,3-BDO into acetic acid after the sugar depletion was also reported by the studies of O‘Hair et al. (2020) and Wang et al. (2003) [14,29], who stated that the 2,3-BDO is most likely converted to acetyl-CoA through reversible pathways, generating acetic acid. Lactic acid and ethanol remained at low levels (<0.7 g/L) throughout the 48 h fermentation, indicating the high specificity of *B. licheniformis* YNP5-TSU to produce 2,3-BDO as the dominant end-product.

When the initial glucose concentration increased to 56.1 g/L, all glucose was consumed after 60 h of fermentation (Figure 1B). The highest concentration of 2,3-BDO occurred at 48 h (18.9 g/L) and the concentration steadily declined afterward. This indicated that glucose might have been completely consumed at a time point between 48 and 60 h and the produced 2,3-BDO was converted to acids when no carbon source was available. Acetic acid concentration slightly increased from 0.37 to 1.73 g/L which is most likely from the consumption of 2,3-BDO. The yield of 2,3-BDO was 0.36 g/g at 48 h of fermentation which was similar to the yield of the fermentation of 38.6 g/L glucose.

When we further increased the initial glucose concentration to 72.9 g/L, the fermentation took longer, and all glucose was consumed between 84 and 96 h (Figure 1C). The highest 2,3-BDO concentration of 23.1 g/L was found at 84 h followed by a slight decrease until 96 h after fermentation started. Lactic acid, acetic acid, and ethanol all remained lower than 1.0 g/L. The yield of 2,3-BDO at 84 h was 0.33 g/g. These results showed that increasing initial glucose concentration from 38.6 g/L to 72.9 g/L only led to a slight decrease (~11%) of 2,3-BDO production yield from 0.37 to 0.33 g/g. *B. licheniformis* YNP5-TSU can continuously use glucose to produce 2,3-BDO without inhibition. Li et al. (2013) reported that when the initial glucose concentration exceeded 152.0 g/L, glucose utilization was inhibited when using *B. licheniformis* 10-1-A [27]. However, a sugar concentration between 64.0 and 125.6 g/L resulted in a successful fermentation with the 2,3-BDO titer being higher than 17.5 g/L. Song et al. (2018) also investigated how different initial glucose concentrations affected the 2,3-BDO production of *B. licheniformis* GSC3102 [23]. Increasing initial glucose concentration from 25 to 100 g/L led to an increased 2,3-BDO titer, while further increasing initial glucose concentration to 150 g/L inhibited 2,3-BDO production. These studies confirmed our results and that under 100 g/L of initial glucose concentration, the 2,3-BDO production is not inhibited. With this data, *B. licheniformis* YNP5-TSU shows it may be a promising microorganism to utilize all glucose for 2,3-BDO production.

However, it is worth noting that our findings also revealed an influence on the sugar consumption rate and 2,3-BDO productivity of the increased initial glucose concentration. For the fermentation with the initial glucose concentration of 56.1 g/L, a total of 42.1 g/L glucose was consumed in the first 36 h compared to only 30.8 g/L glucose being consumed when the initial glucose concentration was 72.9 g/L. The glucose consumption rate decreased from 0.93 to 0.76 g/L/h when the initial glucose concentration increased from 56.1 to 72.9 g/L. The 2,3-BDO productivity was 0.39 g/L/h when the initial glucose concentrations were 38.6 and 56.1 g/L. However, the productivity decreased to 0.27 g/L/h when the initial glucose concentration increased to 72.9 g/L. These results indicated that the initial glucose concentration around 60 g/L might be optimal for *B. licheniformis* YNP5-TSU with a promising production yield of 2,3-BDO and a high 2,3-BDO productivity. Further increasing the initial glucose concentration slowed down the fermentation.

### 3.2. Fermentation of Glucose and Food Waste Hydrolysate Media with Different Yeast Extract and Peptone Concentrations

**Figure 2:**
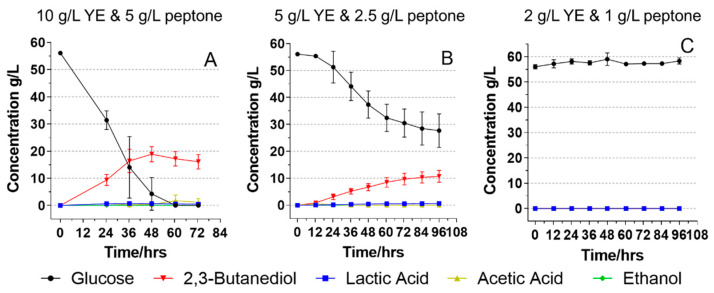
Fermentation of *B. licheniformis* YNP5-TSU using glucose fermentation media (60 g/L) with three different yeast extract and peptone concentrations—10 g/L yeast extract and 5 g/L peptone (**A**); 5 g/L yeast extract and 2.5 g/L peptone (**B**); 2 g/L yeast extract and 1 g/L peptone (**C**). YE: yeast extract.

While nutrients, especially nitrogen, play an essential role in 2,3-BDO fermentation, the added cost can greatly affect the economics of 2,3-BDO fermentation. Figure 2 shows the fermentation results of using glucose media with different yeast extract and peptone concentrations. All three media contained 56.0 g/L glucose, with varying concentrations of yeast extract and peptone. *B. licheniformis* YNP5-TSU was able to consume all glucose within 60 h with a 2,3-BDO titer of 17.2 g/L (Figure 2A) when yeast extract was 10 g/L and peptone was 5 g/L. In the first 24 h, 24.7 g/L of the glucose was consumed, the remaining 27.1 g/L was consumed in the next 24 h. When the yeast extract and peptone contents were respectively decreased by half to 5 and 2.5 g/L, respectively, the fermentation slowed down (Figure 2B). Only 4.8 g/L of the glucose was used in the first 24 h followed by another 13.9 g/L used in the next 24 h. Later than that, less than 10.0 g/L of the glucose was consumed by the microorganisms every 24 h with 27.7 g/L glucose still remained in the media at 96 h. Meanwhile, the productivity of 2,3-BDO decreased significantly from 0.39 to only 0.12 g/L/h (*p* < 0.05).

**Figure 3:**
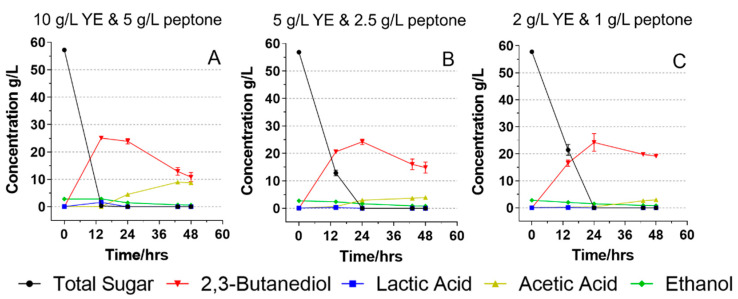
Fermentation of *B. licheniformis* YNP5-TSU using food waste hydrolysate fermentation media with three different yeast extract and peptone concentrations—10 g/L yeast extract and 5 g/L peptone (**A**); 5 g/L yeast extract and 2.5 g/L peptone (**B**); 2 g/L yeast extract and 1 g/L peptone (**C**).

Furthermore, the fermentation did not occur when the yeast extract and peptone contents were 2 and 1 g/L, respectively (Figure 3C). This result indicated that *B. licheniformis* YNP5-TSU was not able to consume any glucose without sufficient nitrogen source or essential nutrients from yeast extract and peptone. Tsigoriyna et al. (2021) have optimized the fermentation parameters for the production of 2,3-BDO using their *B. licheniformis* strain. Different media compositions including yeast extract, tryptone, (NH_4_)_2_SO_4_, KH_2_PO_4_, K_2_HPO_4_, MgSO_4_, ammonium acetate, etc. were investigated [30]. They found that both yeast extract and tryptone had positive effects on the production of 2,3-BDO with yeast extract showing the most influencing effect on the fermentation. For their strain, the optimal yeast and tryptone content in the media were high: 13.4 and 6.4 g/L, respectively. Our results also showed that for the glucose media, there is a high requirement of nitrogen content for maintaining the fermentation capability of the *B. licheniformis* YNP5-TSU. Insufficient nitrogen source leads to the slowdown, and even the failure, of the fermentation.

Since food waste hydrolysates contain a significant amount of nitrogen and essential nutrients (e.g., soluble proteins and amino acids), the high demand for nutrients in 2,3-BDO fermentation may be relieved by using food waste hydrolysate media. To this end, we prepared food waste hydrolysates with different yeast extract and peptone concentrations to investigate if the indigenous nutrients in food waste can potentially reduce the nitrogen requirement and improve 2,3-BDO fermentation. In all media, the initial total sugar concentrations were around 57.0 g/L. When the yeast extract and peptone concentrations were 10 and 5 g/L respectively, almost all sugars were consumed within 14 h with only 0.3 g/L of sugars left (Figure 3A). The highest 2,3-BDO concentration of 25.1 g/L was found at 12 h. This fermentation rate is much higher than that in the fermentation using glucose at the same level of nutrients. This is probably attributed to some other essential nutrients (e.g., minerals and vitamins) that stimulate the growth and metabolisms of microorganisms. Previously, Huang et al. (2015) [5] also found that the food waste media led to an increased fermentation rate to produce butanol compared to the glucose media.

When the yeast extract and peptone content decreased to 5 and 2.5 g/L, the *B. licheniformis* YNP5-TSU was still able to consume all 56.8 g/L of total sugars within 24 h, where the highest 2,3-BDO content of 24.2 g/L was found (Figure 3B). Even with decreased yeast extract (2 g/L) and peptone (1 g/L), 57.8 g/L of total sugars was still consumed by YNP5-TSU in 24 h, producing 24.2 g/L of 2,3-BDO (Figure 3C). The successful fermentation of food waste with low additions of yeast extract and peptone may be due to the 7.8% of protein content in the food waste which provides a potential amino acid source. These findings indicated that food waste used in this study and its enzymatic hydrolysate can be used as a promising feedstock to produce 2,3-BDO by *B. licheniformis* YNP5-TSU without the need for heavy loading of nitrogen sources.

The one downside, however, without sufficient yeast extract or peptone in food waste media, is that the fermentation rate in the first 12 h was slowed down. By reducing the yeast extract and peptone (5 and 2.5 g/L, respectively) by half, the sugar consumption in the first 12 h decreased from 56.9 to 44.0 g/L. Moreover, fewer sugars, 36.3 g/L, were consumed within the first 12 h when using the lowest yeast extract and peptone concentrations (2 and 1 g/L, respectively). For the whole fermentation, the 2,3-BDO productivity decreased significantly from 1.79 to 1.01 g/L/h when yeast extract decreased from 10 to 2 g/L and peptone decreased from 5 to 1 g/L. Although the sugar consumption rate slowed down in the initial 12 h and the overall 2,3-BDO productivity decreased, the 2,3-BDO yield from each gram sugar was not jeopardized by decreasing yeast extract and peptone concentrations. Compared to the glucose media, the food waste and its hydrolysate usually contain substances such as minerals and vitamins which are essential nutrients for microbial 2,3-BDO production [31]. Jurchescu et al. (2013) reported a high production of 2,3-BDO using a rich media with various macro- and micro-elements in addition to glucose [32]. *B. licheniformis* NCIMB 8059, the strain investigated in the study of Białkowska et al. (2015), could not consume any glucose or fructose to produce 2,3-BDO without adding a mineral mixture [31]. However, when using enzymatic apple pomace hydrolysate (10 g/L) as the media, the yield of 2,3-BDO reached as high as 0.47 g/g. Another potential factor could be protein content. As an auxotrophic organism, *B. licheniformis* YNP5-TSU cannot synthesize several amino acids and must obtain them from the environment [33].

### 3.3. Fermentation on Food Waste Hydrolysate with Different Starting Total Sugar Concentrations

**Figure 4:**
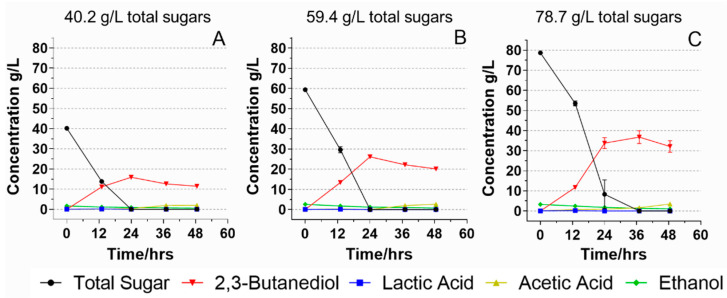
Fermentation of *B. licheniformis* YNP5-TSU using food waste hydrolysate fermentation media with three different initial total sugar concentrations—40.2 g/L (**A**), 59.4 g/L (**B**), and 78.7 g/L (**C**).

Increasing the substrate (sugar) loading has a profoundly positive economic impact on 2,3-BDO fermentation, such as reduced equipment size and decreased energy consumption for downstream distillation [4]. Therefore, food waste hydrolysates with three different initial sugar concentrations were prepared to study how different initial total sugar concentrations affect the 2,3-BDO fermentation. The initial sugar concentrations were 40.2, 59.4, and 78.7 g/L, respectively. All food waste hydrolysates were supplemented with minimal nitrogen nutrients: 2 g/L of yeast extract and 1 g/L of peptone. When the initial sugar concentration was 40.2 g/L, it took 24 h for *B. licheniformis* YNP5-TSU to consume all sugars and produce 15.9 g/L 2,3-BDO with a yield of 0.40 g/g (Figure 4A). When the initial sugar increased to 59.4 g/L, the *B. licheniformis* YNP5-TSU was still able to utilize all sugars within 24 h with 26.1 g/L 2,3-BDO produced and an improved yield of 0.44 g/g (Figure 4B).

When the initial sugar concentration was further increased to 78.7 g/L, all sugars were utilized at 36 h to produce 36.7 g/L of 2,3-BDO. In the first 12 h, only 25.2 g/L sugars were consumed followed by a faster consumption of 45.2 g/L sugars over the following 12 h. Although it took longer to consume all sugars, the yield of 2,3-BDO reached as high as 0.47 g/g which was 93% of the theoretical yield (0.5 g/g) [34] and the 2,3-BDO productivity was 0.99 g/L/h, significantly higher than that in the fermentations with 40.2 and 59.4 g/L initial sugar concentrations (*p* < 0.05). Based on the mass balance, 0.28 g of 2,3-BDO can be produced from 1 g bakery food waste (dry mass) through liquefaction, saccharification, and fermentation. In our previous study, using pepper and pineapple waste, high 2,3-BDO yields of 0.49 and 0.44 g/g, respectively, were observed [1]. Moreover, Figure 4C also showed that *B. licheniformis* YNP5-TSU was able to consume high sugar concentrations (70.4 g/L) in the first 24 h of fermentation, provided there was enough sugar remaining. It is noted that, opposite to the fermentation using the glucose media, increased initial sugar concentrations in food waste hydrolysate led to increased 2,3-BDO yield, overall sugar consumption rate, and 2,3-BDO productivity. This result is significant, as it indicates that using food waste as a substrate supports the high-solid fermentation with an improved fermentation yield and rate. Wang et al. (2010) studied the effects of the initial total sugar concentration of corn molasses on 2,3-BDO production using *K. pneumoniae* SDM [35]. Their results showed that 2,3-BDO production maximized when the initial total sugar concentration was 70 g/L and increasing the initial total sugar content to 80 g/L led to reduced 2,3-BDO production. Białkowska et al. (2015) reported that the 2,3-BDO fermentation failed when they used a rich medium of apple pomace hydrolysate containing 50 g/L sugar, probably due to overwhelmed nutrients that inhibited 2,3-BDO biosynthesis. To summarize, our results support that food waste hydrolysate is a promising alternative feedstock for *B. licheniformis* YNP5-TSU to produce 2,3-BDO efficiently. The appropriate minerals, vitamins, and amino acids it might contain can increase the fermentation efficiency and 2,3-BDO yield.

Previous studies have been conducted to produce 2,3-BDO from different agricultural and food wastes using different microorganisms as summarized in Table 1. For comparison purposes, only batch fermentation results are presented in this summary while fed-batch and continuous fermentation results are excluded. As shown in Table 1, the highest 2,3-BDO yield of 0.48 g/g was achieved using *Klebsiella pneumoniae* PM2 on fermenting oil palm empty fruit bunches hydrolysate with an initial sugar concentration of 46 g/L [36]. However, its 2,3-BDO productivity was relatively low (0.53 g/L/h). Li et al. reported a high 2,3-BDO productivity of 2.6 g/L/h when they fermented corn stover hydrolysate (80 g/L) to 2,3-BDO using *Bacillus licheniformis* X10, but the 2,3-BDO yield was low at 0.39 g/g [37]. Using a high initial sugar concentration of 120 g/L, *Bacillus amyloliquefaciens* 18,025 was able to produce a high 2,3-BDO titer of 42.1 g/L and a high 2,3-BDO productivity of 1.56 g/L/h, however, the yield remained as low as 0.38 g/g [38]. Compared to previous studies, our study of fermenting food waste using *B. licheniformis* YNP5-TSU was able to reach a high 2,3-BDO titer of 36.7 g/L, a high yield of 0.47 g/g, and a high productivity of 0.99 g/L/h under the optimized condition. With its ability to work well under both alkaliphilic and thermophilic conditions, *B. licheniformis* YNP5-TSU showed its unique advantage in producing 2,3-BDO using food waste as feedstock.

More importantly, in our study, only a small amount of nutrients (2 g/L yeast extract, 1 g/L peptone) were supplemented to the fermentation media. This nutrient supplementation level is much lower than the ones in other studies in Table 1, which used at least 5 g/L of yeast extract, peptone, or other nitrogenous sources. It is well known that nutrients, especially nitrogenous sources, play a significant role in fermentation by affecting bacterial growth and activities. Yeast extract and peptone are the two most widely used nutrients for 2,3-BDO fermentation; however, the heavy use of the costly yeast extract and peptone could increase the operating cost of fermentation and potentially prohibit the economic production of 2,3-BDO. Therefore, a low nutrient supplementation to fermentation media has a profound impact on 2,3-BDO production. In our study, the lower yeast extract and peptone supplementation (compared to other studies) are probably because food waste itself contains certain amounts of nutrients, such as protein, amino acid, and minerals.

## 4. Conclusions

This study highlights the effects of the concentration of added nutrients (i.e., yeast extract and peptone) and sugar concentrations on the fermentation performance of *B. licheniformis* YNP5-TSU. With sufficient nitrogen source, the optimal initial glucose concentration of the glucose media was 60 g/L for *B. licheniformis* YNP5-TSU. Further increasing the initial glucose concentration (>70 g/L) led to a reduced sugar consumption rate and 2,3-BDO productivity. An insufficient nitrogen source (2 g/L yeast extract and 1 g/L peptone) caused significantly reduced fermentation performance and even failure of the fermentation. However, when food waste media was used, the fermentation went well, even with a low supplementation of nitrogen source (2 g/L yeast extract and 1 g/L peptone). Opposite to the fermentation using glucose media, the increase of initial sugar concentrations in the food waste media enhanced the sugar consumption rate, boosted the 2,3-BDO yield to 0.47 g/g, and increased the productivity to 0.99 g/L/h. Under an optimal fermentation condition, 0.28 g of 2,3-BDO can be produced from 1 g food waste (dry mass) through liquefaction, saccharification, and fermentation. The findings from this study elucidate the behavior of *B. licheniformis* YNP5-TSU and aid in the design of a more efficient, profitable, and energy-friendly fermentation process to produce 2,3-BDO.

## Figures and Tables

**Table 1:** Microbial production of 2,3-BDO using different microbes and food waste carbon sources.

Strain	Carbon Source	Total Sugar (g/L)	Fermentation Type	Media Supplementation	Titer (g/L)	Yield (g/g)	Productivity (g/L/h)	Refs
Bacillus licheniformis YNP5-TSU	Cabbage	14.2	Shaking Flask	Yeast extract 0.5 (*w/v*); Peptone 0.5 (*w/v*)	6.8	0.48	0.28	[1]
Bacillus licheniformis YNP5-TSU	Brewer’s spent grain hydrolysate	48.2	Shaking Flask	Yeast extract 10 g/L; Peptone 5 g/L	20.4	0.45	0.28	[39]
Bacillus licheniformis X10	Corn stover hydrolysate	80.0	Shaking Flask	Yeast extract, 5 g/L; CSLP, 14.5 g/L; Triammonium citrate, 1 g/L; Sodium acetate, 6.5 g/L; K_2_HPO_4_·3H_2_O, 4 g/L; MgSO_4_·7H_2_O, 0.25 g/L	31.2	0.39	2.6	[37]
Bacillus amyloliquefaciens 18,025	Bakery waste hydrolysate	120.0	Bioreactor	Yeast extract 15 g/L, KH_2_PO_4_ 0.5 g/L, K_2_HPO_4_ 2 g/L, KCl 0.3 g/L and MnSO_4_·H_2_O 0.025 g/L	42.1	0.38	1.56	[38]
Enterobacter ludwigii	Brewer’s spent grain hydrolysate	40.0	Bioreactor	(NH_4_)_2_HPO_4_ 6 g/L; (NH_4_)_2_SO_4_ 7.2 g/L; KOH 0.45 g/L; EDTA 0.51 g/L; MgSO_4_·7H_2_O 0.3 g/L; CaCl_2_·6H_2_O 0.09 g/L; FeSO_4_·7H_2_O 0.022 g/L; MnSO_4_·H_2_O 0.0038 g/L; ZnSO_4_·7H_2_O 0.0075 g/L	16.4	0.41	1.03	[40]
Klebsiella pneumoniae PM2	Oil palm empty fruit bunches hydrolysate	46.0	Bioreactor	Tryptone 5 g/L; Yeast extract 5 g/L; K_2_HPO_4_·3H_2_O 7 g/L; KH_2_PO_4_ 5.5 g/L; MgSO_4_·7H_2_O 0.25 g/L; Na₂MoO₄·2H₂O 0.12 g/L; CaCl_2_·2H_2_O 0.021 g/L	19.0	0.48	0.53	[36]
Paenibacillus polymyxa DSM 365	Wheat straw hydrolysate	98.9	Shaking Flask	Yeast extract 5 g/L; Tryptone 3.5 g/L; (NH_4_)_2_SO_4_ 3.0 g/L; KH_2_PO_4_ 3.5 g/L; K_2_HPO_4_ 2.75 g/L; MgSO_4_ 0.2 g/L; NH_4_ acetate 1.5 g/L; CoCl_2_ 0.05 g/L; 3-(N-morpholino) propanesulfonic acid (MOPS) 10 g/L; Trace element solution 3 mL (per liter)	23.4	0.27	0.28	[41]
Bacillus licheniformis YNP5-TSU	Bakery waste hydrolysate	80.0	Shaking Flask	Yeast extract 2 g/L; Peptone 1 g/L	36.7	0.47	0.99	This study

## Data Availability

The datasets generated for this study are available on request to the corresponding author.
